# Quality of life of depressed and suicidal patients seeking services from traditional and faith healers in rural Kenya

**DOI:** 10.1186/s12955-017-0657-1

**Published:** 2017-05-08

**Authors:** Christine W. Musyimi, Victoria N. Mutiso, Sameera S. Nayak, David M. Ndetei, David C. Henderson, Joske Bunders

**Affiliations:** 1 Africa Mental Health Foundation, Nairobi, Kenya; 2 Vrije Universiteit, Amsterdam, Netherlands; 3 Columbia University, New York, USA; 4 University of Nairobi, Nairobi, Kenya; 5 Boston University School of Medicine, Boston, USA; 6 Harvard Medical School, Boston, USA

**Keywords:** Kenya, Traditional healer, Faith healer, Depression, Suicide, Quality of life

## Abstract

**Background:**

In rural Kenya, traditional and faith healers provide an alternative pathway to health care, including mental health care. However, not much is known about the characteristics of the populations they serve. The purpose of this study was to determine the relationship between depression, suicidal ideation, and socio-demographic variables with Quality of Life (QoL) indicators in a sample seeking mental health services from traditional and faith healers in rural Kenya. Understanding QoL in this sample can help develop mental health policy and training to improve the well-being of this population.

**Method:**

This was a cross-sectional epidemiological survey (*n* = 443) conducted over a period of 3 months among adult patients seeking care from traditional and faith healers in rural Kenya. Data were collected using the Beck Depression Inventory II (BDI-II), Beck Scale for Suicide Ideation (BSS) and WHO Quality of Life Survey- BREF (WHOQOL-BREF), and analyzed using correlation analyses, parametric tests, and regression analyses.

**Results:**

Increasing levels of depression were associated with lower QoL among patients seeking care from traditional and faith healers. BSS scores were significantly negatively correlated with overall, physical, psychological, and environmental QoL, *p* < .05. There was a statistically significant difference between mean scores for overall QoL between depressed (*M* = 2.35, *SD* = 0.76) and non-depressed participants (*M* = 3.03, *SD* = 0.67), *t*(441) = 8.899, *p* < .001. Overall life satisfaction for depressed participants (*M* = 2.23, *SD* = 0.69) was significantly lower than non-depressed participants. Regression analyses indicated that depression, suicidal ideation, and being married predicted lower overall QoL controlling for other variables. Post hoc tests and subgroup analysis by gender revealed significant differences for females only. Depression, and older age predicted lower life satisfaction whereas being self-employed predicted higher life satisfaction, when controlling for other variables.

**Conclusion:**

This study sheds light on correlates of QoL in depressed and non-depressed patients in rural Kenya. Evidence suggests that traditional and faith healers treat patients with a variety of QoL issues. Further research should focus on understanding how these issues tie into QoL, and how these healers can target these to improve care.

## Background

Low and middle income countries (LMICs) often have overburdened mental health systems with limited resources, capacity, and infrastructure [12]. At the same time the global burden of disease of mental illness is on the rise, disproportionally affecting LMICs, where the mental health treatment gap is large and expanding [35, 63]. In this context, individuals in LMICs often seek mental health services through alternative pathways of care in the informal sector, such as traditional and faith healers [12]. In Kenya for example, there is a tremendous scarcity of mental health services with less than 100 psychiatrists practicing in the country, which might compel individuals to seek care from healers who are more readily accessible [33, 42]. Furthermore, there are other social and cultural factors that make these providers more favorable and acceptable to the community than physicians practicing western medicine [2]. Studies from Africa suggest that traditional and faith healers play a key role in providing services to a vast number of mentally ill patients [12, 13, 44]. However, not much is known about patients who seek care from these sources.

In Kenya, although not formally recognized, traditional and faith healers offer a parallel system of mental health-care [42]. A recent mixed-methods study in three informal urban settlements around Nairobi, Kenya found that these healers often treat patients with mental illness using a mixture of counseling, herbal remedies, consultations with the spiritual world, and home-visits to cleanse the environment of bewitching forces [44]. The healers in this sample were frequented by individuals with a variety of mental illnesses ranging from common mental illness such as depression and anxiety to more severe forms of mental illness such as schizophrenia [44]. Depression and current suicidal behavior were co-occurring in this sample, which is unsurprising but troubling, given that suicide is a leading cause of mortality worldwide and is frequently associated with depression [16, 40, 44, 67]. Although this study sheds some light on the types of disorders treated in the informal sector in Kenya, there is relatively little information known about the quality of life (QoL) of individuals seeking care for mental illness in this sector.

Despite the fact that health care research has historically approached health outcomes from a morbidity and mortality perspective, research has shown that QoL is a meaningful tool that can be used to predict disease outcomes and patient well-being [19, 41]. QoL is an important construct that provides an indication of an individual’s subjective well-being in relation to their socio-cultural context and value systems [45, 46]. Understanding the factors that are related to QoL in depressed and suicidal patients is crucial for the development of public health policies that can improve overall health outcomes. If research can accurately capture the domains of QoL that are being affected by depression severity and suicidal ideation, then this presents a valuable opportunity to develop well-informed interventions that specifically target these domains. Unfortunately, the research on this subject in Kenya is scarce.

This is the first study, to our knowledge, that has attempted to capture differences in QoL measures between depressed and non-depressed patients in the informal health care sector. There is a dearth of research in Africa, particularly Kenya specifically related to quality of life for individuals with depression. As Kenya moves towards a better integration of mental health into primary health care [30, 31], it is necessary to mobilize the informal and formal sectors to work in partnership as suggested through a recent study in rural Kenya [47]. Since the data on the treatment-seeking population in the informal sector is limited, it is essential to first gather a holistic understanding of this population in order to identify whether their characteristics are similar or dissimilar to those seeking care from formal psychiatric service centers. Thus results from this assessment study can inform the development of interventions targeted to the needs of this population and aimed at improving specific domains of quality of life. The aim of this study is to determine the relationship between depression, suicidal ideation, and socio-demographic variables with QoL indicators in a sample seeking mental health services from traditional and faith healers in rural Kenya.

## Methods

### Participants and procedures

This was part of a longitudinal study that involved the identification of patients with depression and follow-up over a period of 3 months, after baseline assessments in Makueni County, one of the 47 counties in Kenya. A total of 4081 patients who sought care from traditional and faith healers in four regions of Makueni County between September 2014 to November 2014 were screened for depression by traditional and faith healers [46] in order to receive psychosocial interventions by the same healers. Community Health Volunteers (CHVs) were then invited to screen patients for depression in order to assess the severity and frequency of symptoms of depression as well as to measure the QoL of patients. Permission was sought from traditional and faith healers and their patients at the healers’ shrines/offices before data collection. Only 443 participants (71.7% female) with a mean age of 48.2 (SD = 16.7) agreed to undergo another assessment by a different provider (CHV), translating to a 10% participation rate. The low number was attributed to patients’ lack of sufficient time for a second assessment, over-reliance, confidence and trust for their healers, a feeling of wellness especially if their healer confirmed a negative depression score and a first of a kind study to be conducted in the region, especially for a condition perceived to be a symptom related to laziness or a physical condition (for instance headaches, sleep problems, fatigue, reduced or increased appetite). Interestingly, 34% of patients who were screened positive by the healer and 4% of those who were found to be negative agreed to be reassessed. The huge difference (30%) could have been attributed by the depressed participants’ desire to receive interventions. Those who agreed to participate to be screened by CHVs were once again explained the procedures, confidentiality measures, benefits versus risks and the free will to decline or withdraw from the study without incurring any costs or losing any benefits.

Participants independently completed self-report questionnaires (described below under measures). A community health volunteer was present to provide support and answer any questions. In order to maintain confidentiality, each participant was given a unique identification code. A total of 316 participants met the criteria for a depression diagnosis.

### Ethics and informed consent

Kenyatta National Hospital/University of Nairobi Ethics Review Committee provided ethical approval for this study. All patients who agreed to be screened by a community health volunteer provided informed consent to participate in the study.

### Measures

#### Socio-demographic Information

Data on the socio-demographic characteristics of the participants were collected including age, gender, religious belief, highest level of education, marital status and type of employment.

#### Beck depression inventory II (BDI-II)

The BDI-II [5] is a 21-item self-report scale measuring presence and severity of depression symptomatology. The BDI-II has four response options from 0 to 3, with 3 representing greater severity. In this study, “don’t know” and “refused to answer” were coded as 7 and 8 respectively. The BDI-II total score was calculated by summing up the response options; codes 7 and 8 were excluded from the total score calculation. BDI-II total scores range from 0–63 with a higher score representing increased severity of symptoms. The clinical cut-offs are as follows: 1) 1–10, normal ups and downs; 2) 11–16, mild mood disturbance; 3) 17–20 borderline depression; 4) 21–30, moderate depression; 5) 31–40, severe depression; 6) over 40, extreme depression. In order to characterize the sample into binary groups, BDI-II scores were re-coded into (1) no depression (range 0–20) and (2) yes depression (range 21–63). The BDI-II has high internal consistency, Cronbach’s α = .92 [5]. It has been previously used in Kenya and in other non-western countries [23, 36, 49, 62]. It has been shown to be an effective tool for depression screening in Kenya [49].

#### Beck scale for suicide ideation (BSS)

The BSS [4] is a 21-item self-report scale designed to detect the participant’s desire, attitudes, behaviors, and plans to commit suicide [11]. The first 19 items of the scale are coded with 3 response options from 0 to 2. The first five questions are used to screen for suicidal ideation (SI); if an individual responds affirmatively to any one of these items, the remaining 14 items are then administered. The sum of the first 19 items is calculated to create a total score ranging from 0 to 38. Higher score indicates higher SI. The last items assess incidence and factors related to previous suicide attempts. Internal consistency of the BSS has been shown to be high, Cronbach’s α = .95 [59]. The BSS has been used with a variety of populations [15, 28, 32, 37, 51]. Although it has been used in neighboring Uganda, it has not been used in Kenya prior to this study [50].

#### WHO quality of life survey- BREF (WHOQOL-BREF)

The WHOQOL-BREF [66] is a 26-item scale assessing an individual’s QoL profile. The WHOQOL-BREF assesses four domains: physical, psychological, social relationships, and environment. Furthermore, there are two questions assessing self-reported overall QoL and life satisfaction. Item responses are coded from 1 to 5; items 3, 4, and 26 are reverse coded. Higher scores on each domain indicate a higher quality of life. Mean score of items in each domain is used to calculate a domain score. These scores are then multiplied by 4 in order to make WHOQOL-BREF scores comparable to the longer quality of life tool, WHOQOL-100. Cases with greater than 20% missing data from a domain are automatically excluded from analyses. There were no such cases in this dataset. Internal consistency is acceptable with Cronbach’s α > .70 for physical, psychological, and environment domains but 0.68 for social relationships [56]. The WHOQOL-BREF has been adapted and used cross-culturally in a variety of countries including Kenya, Nigeria, Brazil, Taiwan and Rwanda [1, 6, 22, 34, 48, 68]. The internal consistency ranged from 0.76–0.85 for the Brazilian version and 0.70–0.77 for the Taiwanese version [6, 68]. Similarly, some countries such as Israel, Turkey and Romania have reported marginal Cronbach’s α in the environmental domain [56]. Changes in Cronbach’s α have not been reported in other studies [1, 22, 34, 48]. Furthermore, this tool is derived from the WHOQOL-100 which was developed as part of a large cross-cultural initiative, and hence is likely to provide a relatively robust measure of QoL in different settings [52]. Its equivalence has also been evaluated with a lot of rigor and therefore has the potential to provide valid scores for comparison in different settings [9].

### Data analysis

Double data entry was carried out using IBM SPSS 23. Data was double entered, compared for any discrepancies, corrected and analyzed using the same software. Descriptive statistics were run to determine the demographics of the sample. Independent samples t-tests and Chi-square tests of independence were run to determine if there were significant group differences between depressed and non-depressed participants on these socio-demographic variables. Pearson’s correlation coefficient was performed to determine correlations between scores on the BDI-II, BSS, and WHOQOL-BREF.

Independent samples t-tests were also carried out to determine if there were statistically significant differences on QoL measures for depressed and non-depressed participants. This was repeated to establish differences on QoL measures between participants with SI as compared to those without SI. Multiple regression analyses were then used to determine predictors of QoL for participants in this sample. Dummy variables were created for level of education, employment status, and marital status. A model was run with overall self-reported QoL as the outcome variable and age, gender, depression diagnosis, level of education, employment status, marital status, and SI as predictor variables. This model was then run with life satisfaction, physical QoL, social QoL, psychological QoL, and environmental QoL as outcome variables respectively. Variance inflation factor (VIF) was used to assess multicollinearity between predictor variables in the models. No issues with multicollinearity were found (VIF not larger than 10). Missing data in the analyses were excluded on a case-wise basis. There was no missing data for the BDI-II whereas .2% of BSS had missing data. In regards to the WHOQOL-BREF, there was .5 and .2% missing data for domain 2 (psychological QoL) and domain 3 (social relationships QoL) respectively, while domains 1 (physical QoL) and 4 (environmental QoL) had no missing data. Statistical significance was set at *p* < .05.

## Results

Table 1 includes the socio-demographic characteristics of the sample. Based on clinical cutoffs for the BDI-II, 71.3% of the sample had depression while 28.7% did not. Mean SI was low (*M* = .88, *SD* = 3.837). 94.1% of the sample had no SI on BSS, 5.9% had some SI (endorsed one or more items). Of the depressed sample, 7.9% endorsed SI on the BSS (endorsed one or more items) compared with 0.8% of the non- depressed sample. Significant differences were found between depressed and non-depressed participants in employment status and SI, *p* < .001. Furthermore, participants in each group differed significantly in their choice of healer for treatment, *p* < .001. No other significant differences were found between groups on demographic variables.

**Table 1:** Socio-demographic characteristics of the sample

Variable	Depressed (*n* = 316)	Non-depressed (*n* = 127)	Total (*n* = 443)	Group Differences
Type of healer (%)				
*Traditional healer*	109 (34.5)	1 (0.8)	110 (24.8)	*χ* ^2^ (1, *n﻿* = 443) =55.142, ***p*** **< .001**
*Faith healer*	207 (65.5)	126 (99.2)	333 (75.2)	
Mean age (SD)	48.0 (17.0)	48.8 (15.9)	48.2 (16.7)	t (441) = .490, *p* = .624
Gender (%)				
*Male*	90 (28.5)	38 (29.9)	128 (28.9)	*χ* ^2^ (1, *n * = 443) = .091, *p* = .762
*Female*	226 (71.5)	89 (70.1)	315 (71.1)	
Religion (%)				
*Catholic*	64 (20.3)	32 (25.2)	96 (21.7) ^a^	*χ* ^2^ (4, *n* = 442) =6.614, *p* = .158
*Protestant*	234 (74.3)	86 (67.7)	320 (72.2) ^a^	
*Muslim*	1 (0.3)	0	1 (0.2) ^a^	
*African Traditional Religion*	3 (1.0)	5 (3.9)	8 (1.8) ^a^	
*No official religion*	13 (4.1)	4 (3.1)	17 (3.8) ^a^	
Level of education (%)				
*No formal education*	85 (27.2)	35 (27.6)	120 (27.3) ^b^	*χ* ^2^ (2,* n* = 440) =2.145, *p* = .342
*Primary*	196 (62.6)	73 (57.5)	269 (61.1) ^b^	
*Secondary*	32 (10.2)	19 (15.0)	51 (11.6) ^b^	
Marital Status (%)				
*Single*	32 (10.2)	19 (15.0)	51 (11.6) ^d^	*χ* ^2^ (2, *n* = 441) =2.406, *p* = .300
*Divorced/Separated*	72 (22.9)	31 (24.4)	103 (23.4) ^d^	
*Married/Cohabiting*	210 (66.9)	77 (60.6)	287 (65.1) ^d^	
Employment Status (%)				
*No employment*	159 (50.3)	86 (70.5)	245 (55.9) ^c^	*χ* ^2^ (2,* n* = 438) =19.974, ***p*** **< .001**
*Self Employed*	125 (39.6)	21 (17.2)	146 (33.3) ^c^	
*Employed*	32 (10.1)	15 (12.3)	47 (10.7) ^c^	
Suicidal Ideation (%)	25 (7.9)	1 (0.8)	26 (5.9) ^a^	*χ* ^2^ (1, *n * = 442) =8.356, ***p*** **= .004**

^a^ *n* = 442; ^b^ *n* = 440; ^c^ *n* = 438; ^d^ *n* = 441

Bold are significant values

Pearson’s correlation was carried out between BDI-II scores, BSS scores, WHOQOL-BREF domain scores, and WHOQOL-BREF overall quality and satisfaction scores. Results were in line with previous studies indicating that depression and SI are correlated, and that depression is negatively related to QoL measures, *p* < .001 [27, 53]. A surprising finding indicated that although BSS scores were significantly negatively correlated with overall, physical, psychological, and environmental QoL (*p* < .01), they were not significantly related to overall life satisfaction (*p* = .074) and the quality of social relationships (*p* = .349). Therefore, endorsing SI was linked to reductions in certain domains of QoL, but not all.

There was a statistically significant difference between mean scores for overall QoL between depressed (*M* = 2.35, *SD* = 0.76) and non-depressed participants (*M* = 3.03, *SD* = 0.67), *t*(441) = 8.899, *p* < .001. Overall life satisfaction for depressed participants (*M* = 2.23, *SD* = 0.69) was significantly lower than non-depressed participants (*M* = 2.94, *SD* = 0.77), *t*(427) = 9.289, *p* < .001. Furthermore, depressed participants had significantly lower scores for each of the WHOQOL-BREF domain-specific items than non-depressed participants, *p* < .001.

In line with previous research, depressed participants had much higher SI (*M* = 1.23, *SD* = 4.50) than non-depressed participants (*M* = 0.008, *SD* = 0.09), *t*(440) = -3.063, *p* = .002. Mean scores for overall QoL between suicidal ideating (*M* = 2.12, *SD* = 1.14) and non-suicidal ideating participants (*M* = 2.57, *SD* = 0.76) were lower for suicidal participants, *t*(440) = 2.855, *p* = .005. Furthermore, suicidal ideating participants had significantly lower scores for each of the physical (*p* < .001), psychological (*p* = .007), and environmental (*p* = .010) WHOQOL-BREF domain-specific items than non-suicidal ideating participants. Overall life satisfaction and social relationships QoL were not significantly different between suicidal and non-suicidal ideating participants, *p* = .079 and *p* = .102 respectively.

Predictors included in the regression model were age, gender, level of education, marital status, employment status, depression status, and SI. Age, gender, level of education, marital status, and employment status were included based on previous research, indicating their impact on QoL [3, 22, 25, 34]. Depression and SI were included since they were related to QoL in univariate analyses. This model was run separately with each QoL indicator as an outcome variable.

Table 2 demonstrates the results of the regression analyses for overall QoL and life satisfaction. Significant regression equations were found for both models, *p* < .001. It appears that depression diagnosis, marital status, and SI significantly predict overall QoL, when controlling for covariates. Having a depression diagnosis and SI predict lower overall QoL. Furthermore, being married also predicts lower overall QoL when compared to being single. Life satisfaction in this sample was strongly predicted by age, depression diagnosis, and employment status. Older age and a positive depression diagnosis predicted lower life satisfaction whereas being self-employed predicted higher life satisfaction, when controlling for other variables in the model.

**Table 2:** Multiple regression analyses for overall QoL and life satisfaction as outcome variables

	Outcome Variables							
	Overall Quality of Life F(10,421) = 9.984, *p* < .001				Life Satisfaction F(10,407) = 10.276, *p* < .001			
Predictors	β	SE	t	*p*	β	SE	t	*p*
Age	−.004	.002	−1.889	0.60	−.005	.002	−2.028	**.043** ^*^
Gender	−.016	.080	−.198	.843	−.083	.079	−1.051	.294
Depression Diagnosis	−.697	.080	−8.654	**.000** ^*^	−.739	.081	−9.121	**.000** ^*^
Level of education								
*Primary*	−.058	.089	−.646	.519	.011	.090	.125	.900
*Secondary*	.002	.135	.017	.986	.215	.136	1.587	.113
Marital Status								
*Divorced/Separated*	−.147	.135	−1.085	.278	.225	.136	1.656	.098
*Married/Cohabiting*	−.252	.115	−2.195	**.029** ^*^	−.035	.116	−.306	.760
Employment Status								
*Self Employed*	.208	.081	2.579	.01﻿0*	.167	.081	2.074	.**039** ^*^
*Employed*	.019	.121	.158	.875	.104	.120	.873	.383
Suicidal Ideation	−.021	.009	−2.307	**.022** ^*^	−.008	.009	−.811	.418
	R^2^ = .192				R^2^ = .202			

*p < .05

Bold are significant values

Table 3 shows the results of the regression analyses for each of the four QoL domains measured; physical, psychological, social relationships, and environmental. Significant regression equations were found for each model, *p* < .001. Depression diagnosis predicted a lower QoL in each of the four domains, *p* < .001. Furthermore, older age predicted lower physical QoL but higher psychological QoL. SI was related to lower physical QoL; however, it did not affect any of the other domains. Thus it appears that a depression diagnosis is the most consistent predictor of lowered QoL, across the four domains measured, after controlling for age, gender, level of education, marital status, employment status, and SI.

**Table 3:** Multiple regression analyses for domain-specific QoL as outcome variables

	Outcome Variables							
	Physical QoL F(10,421) = 6.025, *p* < .001		Psychological QoL F(10,419) = 9.807, *p* < .001		Social QoL F(10,420) = 6.975, *p* < .001		Environmental QoL F(10,421) = 8.625, *p* < .001	
Predictors	β	*p*	β	*p*	β	*p*	β	*p*
Age	−.012	**.013** ^*^	.019	**.004** ^*^	.008	.308	.002	.728
Gender	.010	.952	.011	.962	−.195	.490	−.360	.089
Depression Diagnosis	−1.041	**.000** ^*^	−1.888	**.000** ^*^	−2.095	**.000** ^*^	−1.819	**.000** ^*^
Level of education								
*Primary*	.067	.725	.335	.188	.285	.366	.183	.438
*Secondary*	−.142	.625	.286	.463	−.054	.910	.218	.543
Marital Status								
*Divorced/Separated*	.155	.592	.434	.263	.648	.176	.410	.252
*Married/Cohabiting*	−.214	.383	−.253	.443	.141	.727	.273	.369
Employment Status								
*Self Employed*	.142	.410	.114	.620	−.300	.294	.133	.534
*Employed*	.224	.385	.269	.438	−.642	.133	−.151	.637
Suicidal Ideation	−.051	**.010** ^*^	−.038	.152	.012	.709	−.035	.155
	*R* ^2^ = .125		*R* ^2^ = .190		*R* ^2^ = .142		*R* ^2^ = .170	

*p < .05

Bold are significant values

## Discussion

Present research in this population is limited, with a lack of data on the effects of depression and SI on QoL. This study sought to address this gap by elucidating the relationship between depression, SI, and relevant socio-demographic constructs with QoL in a treatment-seeking sample in rural Kenya.

Findings from this sample indicate that depression significantly and adversely impacts all aspects of QoL. These results are consistent with research that has demonstrated a similar relationship between depression and QoL variables [53]. Kenya has limited formal psychiatric infrastructure, and hence traditional and faith healers are able to access a wider range of patients in remote locations, who might ordinarily lack access to care [33, 42, 44]. Therefore, traditional and faith healers are in a unique position to provide intervention services for depression which could result in improving the QoL of individuals in these rural communities. To our knowledge, there are no empirical studies investigating the effects of depression-interventions on QoL indicators in Kenya. However, research from other countries suggests that this might represent a feasible pathway to enhance one of the most important components of health care, QoL [55, 58, 64].

Suicidal ideation was also related to differences in QoL in this sample. Individuals with SI tend to have lower QoL on all domains of this construct [24]. However, in this sample SI predicted decreases in overall QoL and physical QoL but not in the other domains measured. This is in line with a recent study which found that suicidal ideation is related to reductions in physical QoL over time [18]. Physical QoL items in the WHOQOL-BREF are related to daily activities, fatigue, pain, dependence on pharmaceuticals, sleep, mobility, and work capacity [66]. SI has been linked to sleep disturbances, physical pain, and exhaustion/fatigue [10, 14, 29, 54, 57, 61, 65]. Since these facets of functioning are most affected by SI, this could explain the reduced QoL response in this domain for individuals with SI. Findings from this sample suggest that interventions tailored to reduce suicidal ideation should also incorporate methods to alleviate some of the somatic symptoms that might accompany it, such as fatigue. By addressing this symptomology, interventions might make gains both in reduced SI and in improved physical QoL.

Our study indicated that there were interesting socio﻿-demographic characteristics that affected QoL; specifically, age, employment status, and marital status. Increasing age in this sample predicted lower self-reported overall QoL and lower physical QoL but higher psychological QoL. Decreases in physical QoL have been documented in other studies that have examined changes in quality of life over the lifespan [26]. This could be related to overall declining health status with age, which is correlated with reductions in QoL [26]. Moreover, overall decreases in QoL with ageing might be linked to a variety of internal and external factors such as functional disabilities, financial burdens, social isolation, and loneliness [20, 60]. The increases in psychological QoL with increasing age seen in this sample might be associated with more realistic goal-setting and lower standards and aspirations in older cohorts [17]. Research has also shown that older cohorts tend to have less positive and negative affect and emotions, leading to a more stable psychological profile [17]. Moreover, there is evidence to suggest that happiness as a construct has a U-shaped curve, with younger and older cohorts demonstrating higher levels than those in the middle age range [8]. It is possible that the older participants in our cohort fit into this global trend and hence perceive their psychological QoL as higher than their younger counterparts because of heightened happiness. At the same time, it is important to delineate that happiness and QoL are not necessarily the same construct, although they might be related. These results suggest that interventions for depression should factor in addressing somatic complaints for older patients and addressing overall happiness and well-being complaints for middle-aged cohorts. These analyses thus shed light on how depression interventions should be tailored in an age-specific manner in order to be most effective.

Post hoc tests were performed to determine the differences between employment status and depression levels using adjusted residuals to estimate Chi-square value for individual cell and subsequent *P*-values and applying Bonferroni’s correction [21]. Results indicated that the significant differences in depression levels were only found among the self employed and unemployed groups. No significant difference were found among the employed (*p* = 0.509). Furthermore, being self-employed in this sample predicted higher self-reported life satisfaction as compared to being employed or to not working at all. Self-employment is consistently related to both higher job satisfaction and overall life satisfaction [7, 39]. This might be an important avenue to target through livelihoods-based interventions. Interventions directed at promoting entrepreneurship and self-employment could serve as a pathway to foster greater job satisfaction and overall life satisfaction that could in turn achieve a sustainable solution to impoverished populations. However, since this was a cross-sectional study, it is impossible to determine whether symptoms of depression in participants surfaced prior to or during employment.

Lastly, being married or cohabiting with a partner in this sample predicted lower overall self-reported QoL. Our results contradict a recent study in South Korea which found that married participants generally had a higher QoL than other groups [25]. Furthermore, marriage has traditionally been associated with better mental health possibly due to higher rates of social and practical support [3, 38, 43]. However, post hoc tests using Least Significant Difference (LSD) and subgroup analysis by gender revealed significant differences for females only. Even though this might have been accounted for by the higher numbers, further research is needed to establish the validity of this claim in this population.

## Implications

The findings from this study suggest that there is great scope for traditional and faith healers to provide evidence-based care to the community and improve QoL indicators. It appears that in addition to mental illness (depression and SI), marital status, employment status, and age, potentially play important roles in regulating QoL. However, the cross-sectional nature of this study limits interpretations on the causality and reasons for the effects of these predictor variables. Future research would benefit from a focus on longitudinal studies investigating the role of marital quality, age-related physical and psychosocial factors, and changes in employment-status on depressive symptomology and QoL indicators. These results can then be used to develop targeted interventions that address deficits in these indicators to indirectly improve QoL for individuals in rural communities.

## Limitations

There is limited data on the validity and reliability of the measurement tools (BDI-II, BSS, WHOQOL-BREF) in this specific population, and thus results must be interpreted with caution. Furthermore, the cross-sectional nature of this study makes it impossible to infer causality.

## Conclusions

Previous literature has shown that healers in the informal sector treat a variety of mental disorders including affective disorders, psychosis, anxiety disorders, substance use disorders, schizophrenia, and personality disorders [44]. This study builds upon this work to shed light on correlates of QoL in depressed and non-depressed patients in rural Kenya. Evidence suggests that traditional and faith healers provide services for individuals suffering from numerous issues related to QoL. Future research is required to determine how these healers can incorporate care for these issues into their treatment modules.
